# To the Editors of the Medical and Physical Journal

**Published:** 1806-08

**Authors:** 


### *To the Editors of the Medical and Physical Journal.*

GENTLEMEN,

TWO cases of paraphymosis have occurred in my practice since I had the pleasure of last addressing you; both in children, and both happening in the same way by tricks at school. I should certainly have considered them too trifling for your notice, if in one case there had not been retention of urine; by which it became interesting, and of importance. On inspecting the penis in this boy more closely, it was observed, that the orifice of the urethra was literally sealed up; it appeared on further inquiry, that this mischief had been brought on by rough handling, which had caused inflammation, and produced this obstruction, in which, probably, it might be a good deal aided by the stretched frænum bringing the opposite sides of the orifice into close contact. His bladder was much distended, his efforts to unload it were frequent and painful; I punctured with a lancet this barrier, and carefully carried it on until I was certain of having got to the extent of it; he had not immediate power over the bladder, but he was not long ere he got completely relieved by evacuating a large quantity of water. By puncturing also the crystallines below the glans, by a recumbent posture, and by the constant application of cold lotions, the retracted prepuce, in both cases, was brought into its original situation in a few days.

It is my intention to go on with the sequel of any case mentioned here, when any thing occurs that appears to me worth pursuing. The girl, whose case was given in a former statement, had a return of her complaint soon after that was sent you; which was attributed to too great indulgence in eating, joined with a state of costiveness; pain and vomiting returned with less violence than before; to which may be added, a quick pulse, thirst, urine high-coloured and remarkably loaded; swelled belly, uniformly sore, and with evident fluctuation. Leeches and a blister were applied to the abdomen, and pills with calomel and gamboge, and a powder with crem. tart. digitalis and ginger were given, and produced, after a few repetitions, an almost incredible discharge of fæces and urine: and she is now perfectly recovered. The powder was continued twice a day for a week or more, with evident advantage; and when the pulse got down, the warm bath was used every evening for a fortnight.

*June* 10, 1806. A PRACTITIONER in DERBYSHIRE.

